# Infection Screening and Vaccination of Adult and Pediatric Patients with Autoimmune Inflammatory Rheumatic Diseases: An Emirati Delphi Consensus

**DOI:** 10.2174/0115733971368196250117105119

**Published:** 2025-01-21

**Authors:** Ahlam Almarzooqi, Jehad Abdalla, Mohamed Sharif Elsadeg, Noura Zamani, Amel Abdel Gadir Ginawi, Zaid Alrawi, Rajaie Namas, Afra Aldhaheri, Ahmed Zayat, Faisal Elbadawi, Layla ALDabal, Najla Aljaberi, Shazia Abdullah, Suad Hannawi, Khalid A. Alnaqbi, Fatima Al Dhaheri, Beena Hameed, Jamal Al-Saleh

**Affiliations:** 1 Department of Rheumatology, Al Qassimi Hospital, Emirates Health Services, Sharjah, United Arab Emirates;; 2 Department of Infectious Diseases, Sheikh Khalifa Medical City (SKMC), Abu Dhabi, United Arab Emirates;; 3 Pediatric Rheumatology, Al Jalila Children Hospital, Dubai, United Arab Emirates;; 4 Department of Rheumatology, Dubai Hospital, Dubai Academic Health Corporation, Dubai, United Arab Emirates;; 5 Department of Rheumatology, Mediclinic City Hospital, Dubai, United Arab Emirates;; 6 Department of Rheumatology, Clemenceau Medical Center Hospital, Dubai, United Arab Emirates;; 7 Department of Rheumatology, Medical Specialties Institute, Cleveland Clinic Abu Dhabi, Abu Dhabi, United Arab Emirates;; 8 Department of Internal Medicine, Tawam Hospital, Al Ain, United Arab Emirates;; 9 Department of Rheumatology, University Hospital Sharjah, University of Sharjah, Sharjah, United Arab Emirates;; 10 Department of Infectious Disease, Rashid Hospital, Dubai Academic Health Corporate, Dubai, United Arab Emirates;; 11 Department of Pediatrics, College of Medicine and Health Sciences, United Arab Emirates University, Al Ain, United Arab Emirates;; 12 Department of Pediatrics, Sheikh Khalifa Medical City, Abu Dhabi, United Arab Emirates;; 13 Department of Rheumatology, AlKuwait-Dubai hospital (ALBaraha), Emirates Health Services (EHS), Ministry of Health and Prevention (MOHAP), Dubai, United Arab Emirates;; 14 Department of Rheumatology, Tawam Hospital, College of Medicine and Health Sciences, UAE University, Al Ain, United Arab Emirates;; 15 Department of Infectious Disease. College of Medicine and Health Sciences, UAEU, Al Ain, United Arab Emirates;; 16 Department of Internal Medicine, King's College Hospital Dubai, Dubai, United Arab Emirates

**Keywords:** Delphi consensus, infection control, rheumatic diseases, vaccination, pediatric patients, healthcare workers

## Abstract

**Introduction:**

Patients with autoimmune and inflammatory rheumatic diseases (AIIRD) have an increased susceptibility to infections due to their compromised immune systems and the use of immunosuppressive therapies. Infections are a leading cause of morbidity and mortality in these patients, emphasizing the need for strategies such as infection control and vaccination to prevent avoidable harm to both patients and healthcare workers. This study aims to provide expert consensus on infection screening and vaccination guidelines for AIIRD patients.

**Methods:**

A task force of experts from the United Arab Emirates developed a set of statements based on available evidence and expert opinion. The consensus was structured into two main categories: infection screening (9 statements with 23 sub-statements) and vaccination (7 statements).

**Results:**

The infection screening consensus covered nine key areas: tuberculosis (TB) screening (I.1), methods and periodicity of TB screening (I.2), strategies for managing positive IGRA test results (I.3), and infection control for hepatitis B (I.4), hepatitis C (I.5), HIV (I.6), varicella-zoster virus (I.7), and Pneumocystis jirovecii (I.8). The vaccination consensus included recommendations on general vaccination principles (V.0) and specific vaccinations for influenza (V.1), pneumococcal disease (V.2), human papillomavirus (HPV) (V.3), varicella-zoster virus (V.4), tetanus (V.5), and COVID-19 (V.6). Delphi voting showed strong consensus among the task force experts, validating their relevance and applicability for clinicians managing AIIRD patients.

**Conclusion:**

This Emirati consensus provides up-to-date guidance and recommendations for clinicians to enhance the care and safety of AIIRD patients.

## 1 INTRODUCTION

Patients with autoimmune and inflammatory rheumatic diseases (AIIRD) have an increased susceptibility to infections due to their underlying disease and the immunosuppressive therapies used to manage their condition. Infections have been shown to be among the leading causes of morbidity and mortality in these patients, contributing to substantial health, socioeconomic, social, and mental burdens [1, 2]. Therefore, implementing infection control and vaccination strategies is crucial in mitigating complications and curbing the spread of infections in healthcare settings, thus preventing patients and health workers from being harmed by avoidable infections [3, 4]. The importance of implementing strategies for infection control and vaccination is highlighted in the 2022 European Alliance of Associations for Rheumatology (EULAR) [5, 6] and American College of Rheumatology (ACR) [7] recommendations for screening and prophylaxis of chronic and opportunistic infections in adults with AIIRD, along with vaccination guidelines.

The recommendations underscore the importance of shared decision-making, patient education to promptly identify signs and symptoms of infections and periodic reassessment of risk factors for chronic and opportunistic infections. These guidelines also offer insights into screening and prevention, emphasizing how their implementation in clinical practice can effectively reduce the infection risk in AIIRD patients [5-7]. While information on consensus-based approaches to vaccination and infection control for AIIRD patients in the Arab world is limited, studies exploring vaccine hesitancy and acceptance rates in Arab countries shed light on challenges and opportunities. Notably, vaccine hesitancy remains a significant issue in some Arab countries, influenced by factors such as low socioeconomic status, lack of vaccine trust, and misinformation [8, 9].

Nevertheless, some studies have highlighted successful vaccination campaigns in the region, exemplified by the United Arab Emirates (UAE), which has been lauded for its effective preventive measures and free vaccination program [10]. Given the substantial burden of infections in patients with AIIRD and the absence of local guidelines, establishing consensus-based guidance became imperative. This guidance offers a standardized approach to managing AIIRD patients while considering their specific needs in the UAE. By utilizing a Delphi consensus, which gathers real-world clinical insights from multiple experts, the management and follow-up of patients can be improved through a personalized strategy [11, 12]. In this context, Emirati experts in rheumatology, pediatric rheumatology, and infectious diseases convened to develop evidence-based opinions and statements on infection screening/prophylaxis and vaccination for both adult and pediatric AIIRD patients. This methodology ensures that recommendations are attuned to the local context and healthcare system in the UAE, making them pertinent and applicable to healthcare providers and patients in the region while reflecting the latest scientific knowledge. This Emirati consensus could later serve as a model for other countries in the region, aiding them in developing evidence-based guidance for managing AIIRD patients.

## 2 METHODS

**Fig. (1):**
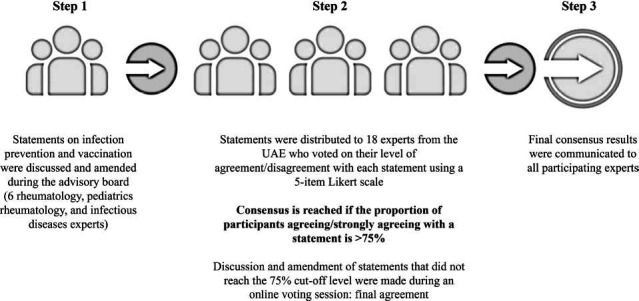
Consensus development methodology.

The Emirati consensus was developed using a Delphi consensus methodology and was completed in three steps (Fig. **1**).

### 2.1 Step 1

A select group of 6 experts from the UAE, comprising adult and pediatric rheumatologists and infectious disease specialists, has been convened to offer specialized insights and recommendations. The involvement of rheumatologists in the development of these guidelines is grounded in their unique expertise in managing autoimmune and inflammatory conditions, coupled with their extensive experience in infection screening and vaccination within various rheumatological contexts. The panel was led by a scientific coordinator chosen for her extensive experience and distinguished record in rheumatology, mainly focusing on the intersection of autoimmune diseases with infection and vaccination, and her history of collaborating on vaccination and screening programs for autoimmune disease patients in her facility. An interactive group discussion was held among experts to review 16 statements initially proposed by the scientific coordinator, supported by the most recent references (Table **1**). These statements were formulated, deliberated upon, and revised by the expert committee as needed, ensuring alignment with scientific evidence and contemporary clinical practices. The authors primarily relied on internationally published recommendations, including those from the EULAR 2022 [5, 6], the American College of Rheumatology (ACR) [7], and the CDC [13-15]. The consensus consisted of two main parts: A- Consensus for the infection screening of patients with AIIRD (9 statements with 23 sub-statements); B- consensus recommendations for the vaccination of patients with AIIRD (7 statements).

Consensus statements related to infection screening and control included the following: I.0- Overarching principles; I.1-Tuberculosis screening; I.2- Methods & periodicity of screening for tuberculosis; I.3- Strategies for a positive IGRA test; I.4- Screening and infection control for hepatitis B; I.5- Screening and infection control for hepatitis C; I.6- Screening and infection control for HIV; I.7- Screening and infection control for Varicella Zoster Virus; I.8. Screening and infection control for *Pneumocystis jirovecii.*

Consensus statements pertaining to vaccination encompassed the following: V.0- General principles; V.1- Influenza vaccination; V.2- Pneumococcal vaccination; V.3- Human papillomavirus (HPV) vaccination; V.4- Varicella-zoster vaccination (VZV); V.5- Tetanus vaccination; and V.6- COVID-19 vaccination and prophylaxis.

### 2.2 Step 2

Before the voting session, the final statements from Step 1 were shared electronically with 18 rheumatology, pediatrics rheumatology, and infectious diseases experts. Each expert rated the level of agreement or disagreement with each statement on a 5-point Likert scale: 1 (absolutely agree), 2 (agree), 3 (neutral), 4 (disagree), and 5 (absolutely disagree) [11, 16]. Consensus was defined as over 75% agreement or disagreement among participants, following guidelines from prior rheumatology research [17]. Statements failing to achieve consensus were designated for further discussion in the next voting session.

### 2.3 Step 3

Following the expert discussions in Step 2, four statements were revised and refined and then voted for final agreement. This manuscript was developed based on the insights and deliberations from the group discussions and was thoroughly reviewed by all participating experts to integrate their collective expertise and perspectives.

## 3 RESULTS AND DISCUSSION

Consensus results and recommendations: strategies for screening and preventing chronic and opportunistic infections in patients with AIIRD.

Out of the 23 sub-statements related to infection screening and control that were sent for voting, only three failed to reach a consensus. These statements were discussed and amended during the voting session (Table **1**).

### 3.1 Overarching Principles: (Adapted from [6])

The following overarching principles articulate the reasoning behind formulating the set of recommendations presented in this article and emphasize crucial concepts in the management of AIIRD. Three principles were formulated and received a high level of consensus by the extended expert panel (94%) (Table **1**).

(A) The risk of chronic and opportunistic infections should be considered and discussed with all patients with AIIRD prior to treatment with csDMARDs (Conventional disease-modifying antirheumatic drugs), tsDMARDs (Targeted synthetic disease-modifying antirheumatic drugs), bDMARDs (Biological disease-modifying antirheumatic drugs, immunosuppressants, and/or glucocorticoids; this risk should be reassessed periodically.

(B) Collaboration is needed between rheumatologists and other specialists, including but not limited to infectious disease (ID) specialists, gastroenterologists, hepatologists, and pulmonologists.

B.1. Rheumatologists bear primary responsibility for treating people with AIIRD. They should work collaboratively with other specialists from other disciplines when planning the prevention or management of chronic and opportunistic infections in patients who receive antirheumatic drugs. The list of available medications for AIIRD in the UAE is presented in Supplementary Table **1**.

(C) Individual risk factors should guide decisions about chronic and opportunistic infection screening and prophylaxis; these risk factors should be reassessed periodically throughout the treatment.

## 4 TUBERCULOSIS

### 4.1 Statement I.1: Tuberculosis Screening (Adapted from the EULAR 2022 [5, 6])

Screening for latent tuberculosis (TB) is recommended before initiating bDMARDs or tsDMARDs. It should also be considered in patients at increased risk of latent TB before starting csDMARDs, immunosuppressants, and/or glucocorticoids (according to the dose and duration).

1. Screening for latent TB is recommended before starting bDMARDS or tsDMARDs.
2. Screening should be considered in patients treated with glucocorticoids depending on the doses, duration of treatment, and risk factors:

**1.2.** Screening should be considered particularly in those patients likely to receive at least 20 mg of prednisolone (or equivalent)/day for more than 2-4 weeks [18]; the treating physician should decide whether their patients are at risk and would benefit from a screening for latent TB: level of agreement of 86%.

This statement was obtained after amending the original statement, which had only a 72% level of agreement: “Screening should be considered, particularly in those patients likely to receive at least 20 mg of prednisolone (or equivalent)/day for more than 2-4 weeks”.

Experts also reached a consensus to eliminate the statement: “For patients who have received doses of prednisolone ranging from 7.5-20 mg (or equivalent)/day for less than three months, the treating physician should decide whether their patients are at risk and would benefit from screening for latent TB”. Instead, they opted to retain the previously voted-upon general statement.

3. Screening is conditionally recommended before initiating csDMARDs based on the clinical assessment of the treating physician and individual risk factors (100% level of agreement). The initial statement was: “Screening should be considered before initiating csDMARDs based on the clinical assessment of the treating physician, individual cases, and clinical conditions”. However, since it achieved only a 67% agreement level, it was modified and voted on in its final version.

4. Physicians are requested not to start treatment before getting the screening results.

### 4.2 Discussion Statement

According to the latest World Health Organization (WHO) tuberculosis report, the estimated total TB incidence rate in the UAE is 0.76 (0.65-0.87) per 100,000 population [19], with an increase of 0.1 cases per 100,000 population in 2021 compared to previous years [20]. The UAE is considered an endemic area for TB, with a significant number of foreign workers coming from regions where TB is prevalent. A noteworthy study from the Health Authority in Abu Dhabi revealed a prevalence of pulmonary TB disease of 39 per 100,000 in the “visa screening program,” with new applicants more likely to test positive than renewals [21]. Consequently, experts emphasize the importance of screening for latent TB in patients before initiating certain medications, particularly bDMARDs or tsDMARDs, and conditional consideration before treatment initiation with csDMARDs, immunosuppressants, and/or glucocorticoids, based on dose and duration [18].

A critical discussion point among the expert team revolved around determining the threshold dose and duration of glucocorticoids that would warrant systematic screening for latent TB. Some experts argued that the dose of 20 mg of prednisolone (or equivalent)/day is relatively low in AIIRD patients, as most patients receive higher doses. Concerns were raised that applying this stringent cutoff would necessitate screening of almost all patients, posing challenges in daily practice. However, the final consensus was to maintain the dose of 20 mg of prednisolone (or equivalent)/day for more than 2-4 weeks, aligning with the suggested minimal dose for glucocorticoid immunosuppression in most guidelines [18]. It was also agreed to emphasize that the treating physician should ultimately decide whether their patients are at risk and would benefit from screening for latent TB.

### 4.3 Statement I.2: Methods & Periodicity of Screening (Adapted from the EULAR 2022 [5, 6])

Screening for latent TB should typically include a chest X-ray and Interferon-gamma release assay (IGRA).

1. In addition to chest X-ray, IGRA (TB QuantiFERON gold test) should be preferred over Tuberculin Skin Test (TST) for TB screening.
2. Periodicity of screening: “There is no consensus nor robust data for how often periodic prescreening should be performed. The IGRA test might be assessed periodically, depending on the clinical judgment of the physician and any change in the patient’s clinical condition”, agreement level of 89%.
3. During the decision-making process, authors were presented with two statements: the initial one, which achieved the best voting results, and the statement below, which failed to reach a consensus and was consequently omitted from the guidelines due to an agreement level of only 56%: “There is no consensus nor robust data for how often periodic prescreening should be performed. The IGRA test might be assessed every 12-18 months, depending on the clinical judgment of the physician and any change in the patient’s condition”.
4. IGRA TB QuantiFERON test is performed for the screening of latent TB in the pediatric population. However, limitations are noted below some group ages (3 or 5, years, depending on references). Referral to ID specialists is warranted.

### 4.4 Discussion Statement

During the voting session, discussions revolved around whether to set a specific timeframe for the periodicity of TB screening. However, given the lack of robust data on this matter [5, 6], experts reached a consensus that the assessment frequency should be left to the discretion of the physician and contingent upon any changes in the patient’s clinical condition. Additionally, some experts proposed the removal of chest X-rays for TB screening, advocating for the sole use of the IGRA test. Despite the absence of robust evidence supporting the utility of X-rays, as indicated in the latest EULAR guidelines [6], the final consensus favored retaining it in alignment with international recommendations. This decision was particularly emphasized, considering that a negative IGRA or TST result cannot conclusively rule out active TB or latent TB [6, 22].

### 4.5 Statement I.3: Strategies for a Positive IGRA Test (Adapted from the EULAR 2022 [5, 6])

In case of a positive IGRA test, the patient should be assessed for latent or active TB, as indicated in Table **2** and the algorithm in Fig. (**2**).

1. If latent or active TB is diagnosed, then the patient should be referred to an ID specialist or pulmonologist for further assessment and should start treatment with particular attention to the interaction with medications used to treat AIIRD.
2. It is not recommended that TB be managed by rheumatologists; patients should be referred to specialists.
3. If latent TB is diagnosed, treatment for TB should be initiated, and biological treatment can be started after four weeks (Treatment regimens are presented in Table **3**; Treatment scheme options for patients with latent tuberculosis therapy are summarized in Supplementary Table **2**).

### 4.6 Discussion Statement

**Fig. (2):**
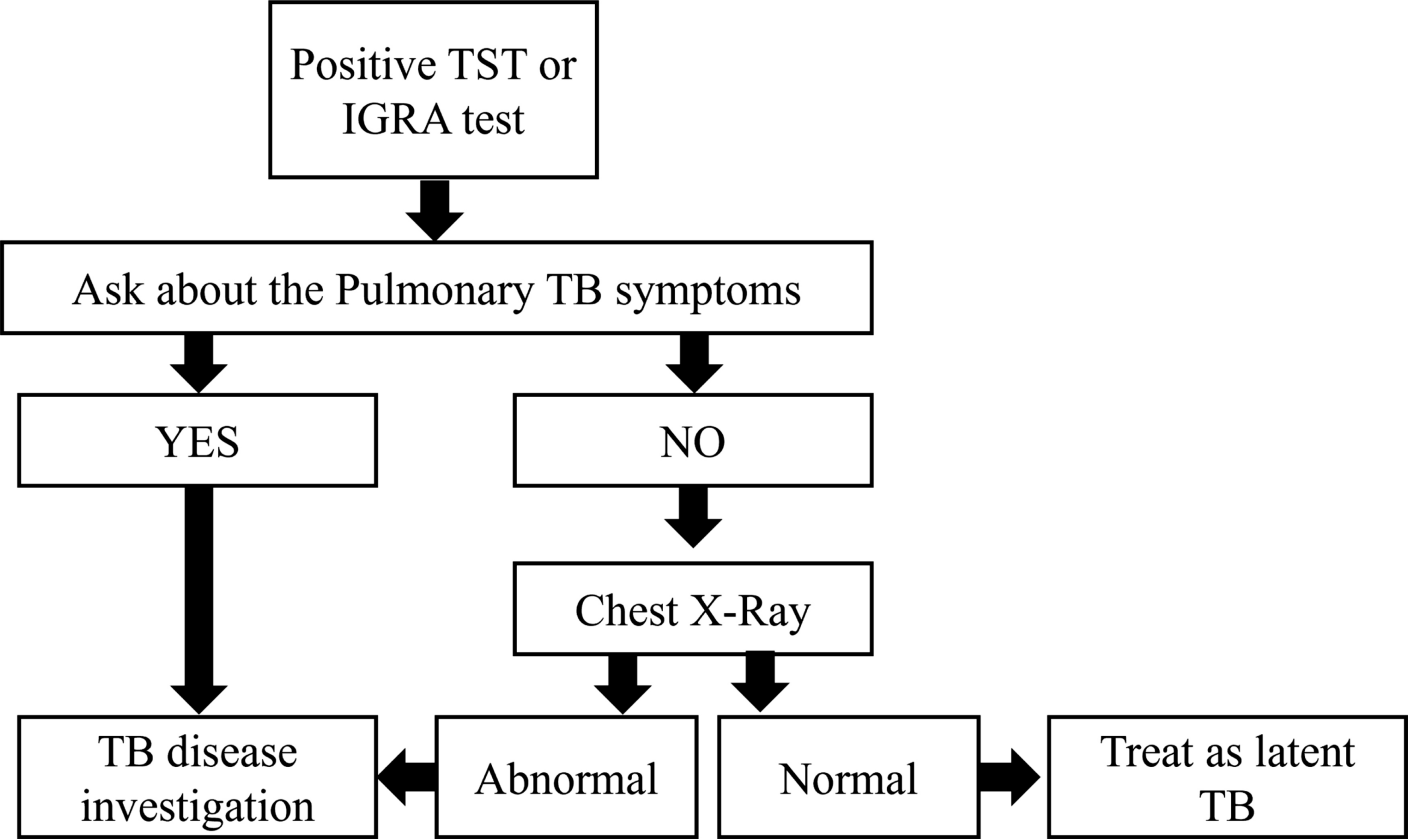
Algorithm for assessment and referral of patients with tuberculosis (TB).

The consensus discussions included strategies for managing a positive IGRA test, and the final consensus incorporated summary tables and algorithms (Tables **2** and **3** and Fig. **2**). These tools were introduced to assist rheumatologists who may not have easy access to a pulmonologist or ID specialist. Nonetheless, a unanimous agreement among experts was that the management of TB should not fall within the purview of rheumatologists. Instead, patients with positive test results should be promptly referred to specialists for appropriate and specialized care.

## 5 HEPATITIS B (STATEMENT I.4) (ADAPTED FROM THE EULAR 2022 [5, 6])

All patients considered for treatment with bDMARDs, tsDMARDs, immunosuppressants, and glucocorticoids (20 mg of prednisolone (or equivalent)/day for more than 2-4 weeks) should be screened for HBV.

A previous statement included csDMARDs along with other treatments. However, during the voting session, experts agreed to remove csDMARDs from the statement for better feasibility and to ensure the early initiation of treatment within cost constraints, resulting in an 85% vote agreement.

1. The risk of HBV reactivation should be assessed before starting csDMARDs, according to the treating physician’s judgment and on an individual, case-by-case basis.
2. Typical screening for hepatitis B virus (HBV) status includes hepatitis B surface antigen (HBsAg), hepatitis B core antibody (anti-Hbcore), and hepatitis B surface antibody immunity (anti-HBs).
3. HBV status should be determined before initiating the treatment for AIIRD and deciding whether vaccination is needed. HBV status reflects that the patient may be:
  - Unexposed (Table **4**)
  - Vaccinated
  - Carrier (HbsAg-positive)
  - Resolved (anti-Hbcore-positive and HbsAg-negative).
4. Table **4** summarizes the suggested procedures based on HBV tests and status.

The risk of HBV reactivation should be assessed on a case-by-case basis for patients likely to receive at least 20 mg of prednisolone (or equivalent)/day for more than 2-4 weeks [18].

Antiviral therapy should start ideally before or at least simultaneously with the treatment for AIIRD and continue for at least 6-12 months after discontinuing antirheumatic treatment.

Periodic testing (adult population): There is a lack of data on when to re-screen for HBV reactivation. Periodic testing should be performed based on individual evaluations, considering risk factors and costs. Referral to hepatologists is also recommended.

Periodic testing (pediatric population): If the screening for HBV is performed before initiating treatment with bDMARDs, periodic testing will not be repeated unless the treating physician decides otherwise (*e.g*., after blood transfusion, if liver enzymes are abnormal, or if other risk factors are identified).

### 5.1 Discussion Statement

The current consensus recommends a comprehensive screening for Hepatitis B virus (HBV) in patients considered for treatment with bDMARDs, tsDMARDs, immunosuppressants, and glucocorticoids. During the voting session, experts concurred to exclude screening recommendations for patients treated with csDMARDs, citing studies indicating a low risk of reactivation with these treatments [23, 24] and recognizing the potential cost implications for both the patients and the healthcare system. Consequently, reactivation assessments should be conducted at the discretion of the clinician on a case-by-case basis. The task force has also developed a guidance algorithm for decision-making in the context of managing HBV reactivation risk. The algorithm provides a structured approach to screening, determining HBV status, and outlining management strategies based on individual patient profiles. The suggested procedures based on HBV testing and status include referral to specialists for antiviral prophylactic treatment in carriers, resolved cases, and those at risk of reactivation. This comprehensive algorithm aligns with the latest EULAR recommendations, emphasizing the significance of a systematic and individualized approach in clinical practice [6].

## 6 HEPATITIS C (STATEMENT I.5) (ADAPTED FROM THE EULAR 2022 [5, 6])

The statement related to HCV achieved an 89% agreement level. It comprised the following:

Screening for chronic hepatitis C (HCV) should be considered before initiating csDMARDs, bDMARDs, tsDMARDs, immunosuppressants, and glucocorticoids (according to dose and duration). Screening is recommended for patients with elevated alanine aminotransferase or those with known risk factors.

1. Screening for HCV is mandatory before starting bDMARDs and tsDMARDs.
2. Screening for HCV should be done before starting prednisolone at doses of at least 20 mg of prednisolone (or equivalent)/day for more than 2-4 weeks [18].
3. Screening for HCV should be done on a case-by-case basis before starting csDMARDs.
4. Screening for HCV includes anti-HCV antibodies; if these are present, the patient should be referred to a hepatologist or ID specialist for further testing/evaluation and treatment.

### 6.1 Discussion Statement

Several studies investigating HCV reactivation in the context of AIIRD treatment, particularly with TNF inhibitors, indicate that it occurred in a small number of patients, and most were conducted before the availability of newer, more effective HCV drugs [5, 25]. The expert panel suggests considering screening for AIIRD patients before treatment initiation, especially with bDMARDs and tsDMARDs, particularly in patients with concurrent HCV risk factors or abnormal liver function tests. Additionally, the Emirati task force recommends HCV screening before initiating glucocorticoids, acknowledging the absence of data in international guidelines and emphasizing the need for context-specific considerations. In June 2023, the Dubai Academic Health Corporation launched a three-year hepatitis C awareness campaign as part of the “EKSHEF” screening program for the early detection of both communicable and non-communicable diseases. This initiative underscores the importance of early detection and prevention, aiming to eliminate hepatitis C by 2030 [26]. Furthermore, the Dubai Academic Health Corporation initiated the Hepatitis C Patient Support Program (HCV PSP) to eradicate HCV in Dubai by offering screening and treatment, particularly for insured residents with existing or newly detected HCV infection [27]. These comprehensive guidelines and programs emphasize the crucial role of early detection and treatment in preventing liver disease progression, aligning with global efforts to control and manage hepatitis C infections.

## 7 HUMAN IMMUNODEFICIENCY VIRUS (HIV) (STATEMENT I.6) (ADAPTED FROM THE EULAR 2022 [5, 6])

Screening for HIV is considered based on high-risk situations assessed by the treating physician.

1. Screening for HIV *via* HIV-Ag (1+2) should be done once before treatment with bDMARDs and tsDMARDs (conditional); if the test is positive, the patient should be referred to an ID specialist.
2. No screening is recommended for pediatric populations.

### 7.1 Discussion Statement

In the absence of robust data regarding the safety of DMARDs, immunosuppressants, or glucocorticoids in HIV-positive patients [5, 6], and considering the cost-effectiveness of screening, the expert panel advocates for HIV screening before bDMARD treatment and conditionally before tsDMARDs, with appropriate care provided if indicated.

## 8 VARICELLA ZOSTER VIRUS (STATEMENT I.7) (ADAPTED FROM THE EULAR 2022 [5, 6])

All patients who are non-immune to Varicella Zoster virus (VZV) and are starting csDMARDs, bDMARDs, tsDMARDs, immunosuppressants, and/or glucocorticoids should be informed about post-exposure prophylaxis following contact with VZV (94% agreement level).

1. No serological screening for VZV immunity is recommended for patients before starting csDMARDs, bDMARDs, tsDMARDs, immunosuppressants, and/or glucocorticoids.
2. Vaccination should be offered to all patients before initiating tsDMARDs and bDMARDs to prevent the disease or its complications.
3. Vaccination is recommended with the non-live recombinant subunit adjuvant zoster vaccine (cf. section V.4.2).
4. Any physician, including rheumatologists or ID specialists, can recommend and administer the vaccine.

### 8.1 Discussion Statement

Acknowledging that the status of VZV immunity can be influenced by various factors, including access to testing, previous vaccination, or infection history, the expert panel suggests that serological screening for VZV immunity before initiating any treatment is not recommended. Nevertheless, the experts unanimously agreed on the importance of recommending vaccination to prevent the disease or its complications; this recommendation was endorsed by both infectious disease specialists and rheumatologists. These statements received a 94% level of agreement.

### 8.2 *Pneumocystis jirovecii* (Statement I.8)

Prophylaxis against *Pneumocystis jirovecii* pneumonia (PCP) should be considered in patients with AIIRD in whom high doses of glucocorticoids (at least 20 mg of prednisolone (or equivalent)/day for more than 2-4 weeks [18]) are used, especially in combination with immunosuppressants and depending on the risk-benefit ratio and according to the physician’s judgment and clinical condition.

1. The prophylaxis scheme should be decided based on a shared discussion between the rheumatologist and ID specialist, depending on the patient’s condition and associated comorbidities.
2. The main prophylaxis scheme is trimethoprim/sulfamethoxazole (TMP-SMX) 480 mg/day (single-strength) or 960 mg three times a week [6] (Table **5**; Supplementary Table **3**).
3. Other alternative regimens, equally efficacious and with fewer adverse effects, can also be suggested (Table **5**; Supplementary Table **3**).
4. In case of allergy or reported side effects to TMP-SMX, alternative prophylactic medications can be suggested depending on the availability and accessibility in the different institutions. The first suggested option is dapsone. Other alternatives include atovaquone and nebulized pentamidine.
5. Atovaquone should be considered the preferred option over TMP-SMX for patients with Systemic Lupus Erythematosus (SLE) who are at a higher risk of experiencing allergic reactions or lupus flares. The use of TMP-SMX in such cases can lead to serious consequences, including severe lupus flares and allergic reactions [28, 29].
6. Atovaquone should be considered the preferred option over TMP-SMX, specifically in Caucasian patients, individuals with lymphopenia, and those who test positive for anti-SSA antibodies. This subgroup of patients faces a higher risk of experiencing mixed reactions when TMP-SMX is administered [30].

### 8.3 Discussion Statement

The expert panel concurs on the critical need for PCP prophylaxis in AIIRD patients receiving high doses of glucocorticoids and immunosuppressants. While TMP-SMX stands as the primary prophylaxis scheme, alternative regimens like dapsone, atovaquone, and nebulized pentamidine can be considered based on individual patient conditions and associated comorbidities. Atovaquone is recommended over TMP-SMX for specific patient subgroups. The task force has summarized treatment regimens for PCP prophylaxis, including doses and selected adverse events, in adults and adolescents (cf. Supplementary Table **3**), aiming to guide clinicians in their management strategy. The guidelines underscore the significance of tailored prophylaxis regimens for diverse patient populations, stressing the need for shared discussions between rheumatologists and infectious disease specialists based on patient conditions and comorbidities. These statements achieved a 94% level of agreement among experts.

Results of the consensus and recommendations: Vaccination in patients with AIIRD.

The seven statements related to vaccination reached the agreement threshold of 75% (Table **6**).

## 9 GENERAL STATEMENT (STATEMENT V.0)

Vaccine recommendations might change based on updated Centers for Disease Control and Prevention (CDC)/ Advisory Committee on Immunization Practices (ACIP) guidelines. Available vaccines in the UAE are presented in Supplementary Table **4**.

Supplementary Table **5** outlines the recommended timeline between immunosuppressive medication and live-attenuated virus vaccines.

## 10 INFLUENZA VACCINATION (STATEMENT V.1)

1. The influenza vaccine is strongly recommended yearly for patients with AIIRD undergoing immunosuppressive therapy.
2. Rheumatologists should review vaccination history annually. Table **7** presents a summarized guideline for the administration of the influenza vaccine and other non-live attenuated vaccines [32, 33].

### 10.1 Discussion Statement

The expert committee reached a unanimous agreement of 100%, strongly recommending annual influenza vaccination for AIIRD patients undergoing immunosuppressive therapy. This recommendation aligns with the latest ACIP guidelines, which recommend routine annual influenza vaccination for all individuals aged ≥6 months without contraindications, including those who are immunocompromised [34]. Additionally, experts highlighted several considerations regarding the timing of vaccination based on the patient's treatment for their rheumatic condition.

vaccination for influenza [31]. A recent multicenter, prospective, randomized, parallel-group noninferiority trial has shown that a temporary discontinuation of MTX for 1 week after vaccination was non-inferior to a discontinuation of MTX for 2 weeks after vaccination, regarding induction of a satisfactory vaccine response to a seasonal influenza vaccine in patients with RA receiving a stable dose of MTX [33].

## 11 PNEUMOCOCCAL VACCINATION (ADAPTED FROM [7, 15])

1. Physicians should check the CDC guidelines for pneumococcal vaccination due to the rapid changes in this area [7, 15].
2. The pneumococcal vaccine should be administered to AIIRD patients over 65 years receiving immunosuppressive therapy.
3. The most recommended regimen for the pneumococcal vaccination would include the administration of the PCV13 followed by the PPSV23 ≥5 years since the last vaccination dose. Other regimens are also available as recommended by the CDC.
4. PCV20 recommendations
  •   V.2.4.1. For those who have not previously received any pneumococcal vaccine, the CDC recommends the administration of one dose of PCV15 or PCV20:
    -   If PCV15 is used, it should be followed by a dose of PPSV23 at least one year later. The minimum interval is eight weeks and can be considered in adults with an immunocompromising condition, cochlear implant, or cerebrospinal fluid leak.
    -   If PCV20 is used, a dose of PPSV23 is not indicated.
  •   V.2.4.2. For those who have only received PPSV23, the recommendation for one dose of PCV15 or PCV20 is as follows:
    -   The PCV15 or PCV20 dose should be administered at least one year after the most recent PPSV23 vaccination.
    -   Regardless of whether PCV15 or PCV20 is given, an additional dose of PPSV23 is not recommended in those persons since they already received it.
  •   V.2.4.3. For those who have only received PCV13, the CDC recommends the administration of either one dose of PCV20 at least one year after PCV13 and one dose of PPSV23; the minimum interval between the PCV13 and PPSV23 doses will vary depending on their specific risk factor.
5. In patients with an immunocompromising condition (at least eight weeks after PCV13), the CDC recommends the administration of up to two additional doses of PPSV23 in their lifetime. The second dose of PPSV23 should be administered at least five years after the first dose of PPSV23. If a patient is 65 years or older at the time of the second dose, then a third dose is not indicated. For patients who have received PCV13 and one dose of PPSV23, the CDC recommends the administration of either one dose of PCV20 at least five years after the last pneumococcal vaccine or a second dose of PPSV23 at least eight weeks after PCV13 and five years after PPSV23 if they have an immunocompromising condition.

### 11.1 Discussion Statement

Pneumococcal vaccination is strongly recommended for AIIRD patients due to their increased risk of invasive pneumococcal disease and nonbacteremic pneumonia [35, 36]. Both the ACR and EULAR societies advocate for pneumococcal vaccination in most AIIRD patients [7, 31]. The expert panel suggests referring to CDC guidelines for pneumococcal vaccination due to the rapid changes in this area. In summary, experts agree that careful consideration of vaccine type and timing should be based on an individualized assessment of infection risk.

## 12 HUMAN PAPILLOMAVIRUS (HPV) VACCINATION

1. For patients with AIIRD aged between 26 and 45 and receiving immunosuppressive medication and not previously vaccinated, vaccination against human papillomavirus (HPV) is conditionally recommended (optional) based on a shared clinical decision between the physician and the patient.
2. Clinicians are advised to follow the national vaccination guidelines in the UAE for adult and pediatric populations.

### 12.1 Discussion Statement

Addressing the HPV vaccination issue in the UAE is complex due to cultural differences and the stigma linked to sexually transmitted infections. Consequently, experts conditionally recommend it for AIIRD patients aged 26 to 45 on immunosuppressive medication, provided they haven't received prior vaccination. The decision should be made collaboratively between the physician and the patient. Notably, Abu Dhabi has pioneered the incorporation of the HPV vaccine into its immunization program since 2008. However, studies indicate that acceptance and coverage are influenced by cultural factors and awareness. Regional data reveal that university Emirati men's acceptance of the HPV vaccine is around 37%, with an alarmingly low vaccination rate of only 3.1% [37]. Family members of those vaccinated also showed a low vaccination level of 13% [37]. Despite these challenges, experts unanimously emphasize the importance of clinicians following the national vaccination guidelines in the UAE for both adult and pediatric populations. Continuous efforts are needed to enhance education and awareness to improve HPV vaccination rates in the region and consequently reduce the incidence of cervical and other anogenital cancers in the UAE [38].

## 13 VARICELLA-ZOSTER VACCINATION (VZV) (ADAPTED FROM [31])

1. The recombinant varicella-zoster vaccine (VZV) is strongly recommended for patients with AIIRD aged 18 years and above receiving immunosuppressive medication.
2. The type of vaccine and suggested regimen are summarized in Table **8**.
3. It is unnecessary to stop immunosuppressive medication before administering the vaccine.
4. Antipyretic medication (*e.g*., paracetamol/acetamino- phen) is not recommended before vaccination.

### 13.1 Discussion Statement

While the literature lacks specific publications addressing recombinant VZV vaccination in AIIRD patients, it is noteworthy that this vaccine has demonstrated both safety and efficacy in immunosuppressed individuals. The expert panel strongly endorses VZV vaccination in alignment with the ACIP and the latest ACR 2022 guidelines [7, 13].

## 14 TETANUS VACCINATION

1. For patients with AIIRD taking immunosuppressive medication, it is generally considered safe to administer a diphtheria/tetanus vaccine booster, particularly for all adults who sustain dirty wounds and are either unvaccinated or have not received a primary series of tetanus toxoid-containing vaccines. In addition to the vaccine, it is also recommended to administer tetanus immunoglobulin (TIG) for prophylaxis [39].
2. The recommended dose of TIG for prophylaxis is 250 IU, administered intramuscularly.

### 14.1 Discussion Statement

The expert panel strongly recommends tetanus vaccination, substantiated by findings from a prospective multi-center cohort study assessing the safety and immunogenicity of tetanus/diphtheria vaccination in patients with rheumatic diseases [39]. The authors affirm that vaccination is both safe and effective, with no observed reactivation of the disease and no necessity to interrupt ongoing immunosuppressive treatment.

## 15 COVID-19 VACCINATION AND PROPHYLAXIS

1. Patients with AIIRD should receive a COVID-19 vaccine unless contraindicated [32].
2. The mRNA vaccine is recommended in AIIRD patients who are not yet vaccinated.
3. For AIIRD patients receiving any immunosuppressive or immunomodulatory therapy who have already received two doses of mRNA vaccine, an additional dose is recommended ≥28 days after the end of the vaccine series.
4. It is not recommended for healthcare providers to routinely request laboratory tests (*e.g*., antibody tests for IgM and/or IgG against spike or nucleocapsid proteins) to assess immunity to COVID-19 post-vaccination or the need for vaccination in an as-yet-unvaccinated person.
5. AIIRD patients not receiving immunomodulatory treatments should take the first dose of the COVID-19 vaccine before initiating immunomodulatory therapy when feasible.
6. AIIRD patients at high risk for poor outcomes related to COVID-19 should receive monoclonal antibody therapy preventively (pre-exposure prophylaxis).
7. AIIRD patients can be offered long-acting monoclonal antibodies with confirmed efficacy for pre-exposure prophylaxis against COVID-19 in case the circulating variants of concerns are still neutralized. This recommendation applies even to patients who have been vaccinated against COVID-19, are not currently infected with SARS-CoV-2, have not had known recent exposure to an infected individual, and those who have moderate to severe immune compromise, such as patients with AIIRD or undergoing treatments and who may not develop a sufficient immune response to COVID-19 vaccination.
8. AZD3152 (Evusheld 2.0) is an investigational, long-acting antibody that has been developed to have broad neutralizing activity across SARS-CoV-2 strains. Current evidence has shown that compared with Evusheld, ASD3152 (Evusheld 2.0) exhibits higher efficacy and a more prolonged activity.
9. Refer to national guidelines for detailed recommendations regarding prophylaxis and treatment of COVID-19.

### 15.1 Discussion Statement

The expert panel grounded their recommendations on the latest ACR guidelines [32], affirming that the existing literature strongly supports COVID-19 vaccination for patients with AIIRD. They emphasize the safety and efficacy of available vaccines and highlight the significance of prioritization, timing, and education to ensure optimal protection against COVID-19 for this patient population. Supplementary Tables **6** and **7** provide guidance on vaccine dosing, immunomodulatory therapy, and EVUSHELD prophylaxis in patients with AIIRD. Additionally, they advise referring to national guidelines for detailed recommendations on COVID-19 prophylaxis and treatment.

## 16 RESPIRATORY SYNCYTIAL VIRUS (RSV)

Respiratory syncytial virus (RSV) poses a significant threat, causing acute respiratory infections in temperate regions during autumn and winter and in tropical regions during rainy seasons [40, 41]. In older adults or individuals with pre-existing conditions, RSV infection can lead to lower respiratory tract disease, exacerbating underlying health issues and potentially resulting in hospitalization and mortality [42-45].

The RSVPreF^3^ OA vaccine (AREXVY) is on the verge of gaining approval in the UAE. Its single intramuscular (IM) injection of 120 μg RSVPreF^3^, adjuvanted with AS01E - an inactivated vaccine - received FDA approval following a Phase 3, randomized, placebo-controlled, multi-country study [46]. This study demonstrated the efficacy and safety of both single and annual revaccination doses. The vaccine effectively prevented RSV-related acute respiratory infections, lower respiratory tract diseases, and severe RSV-related lower respiratory tract diseases in adults aged 60 years and older [46]. Importantly, this protection applies irrespective of the RSV subtype and the presence of underlying coexisting conditions. It is crucial to note that the vaccination was not initially part of Delphi's original recommendations, as it is currently undergoing the approval process in the UAE. Nevertheless, given its demonstrated effectiveness, it can be considered for recommendation in patients who are 60 years old and older with AIIRD [46].

## CONCLUSION

In summary, this manuscript presents the first consensus guidelines for real-world infection screening and control in the UAE and the Arab world for the treatment of AIIRD patients. These guidelines provide comprehensive guidance for clinicians in making informed decisions on infection screening and prophylaxis, including vaccination. Optimal shared clinical decisions with patients require effective collaboration between rheumatologists/pediatric rheumatologists and infectious diseases, internal medicine, and gastroenterology specialists. Notably, the continuous updating of these guidelines, informed by evolving evidence-based data, is imperative for maintaining their relevance and applicability in the dynamic landscape of medical knowledge.

## Figures and Tables

**Table 1:** Emirati consensus recommendations for the screening and prevention of chronic and opportunistic infections in adults with autoimmune inflammatory rheumatic diseases (adapted from the EULAR 2022 [5, 6] and the American College of Rheumatology (ACR)).

**-**	**Statements**	**Level of Agreement**
**Statement 0:** **General Statement**	**Overarching Principles**	**94%**
**Statement 1:** Tuberculosis screening	**Tuberculosis** 1- Screening for latent tuberculosis (TB) is recommended before initiating bDMARDs or tsDMARDs. It should also be considered in patients at increased risk of latent TB before starting csDMARDs, immunosuppressants, and/or glucocorticoids (according to the dose and duration).	**100%**
	1.1. Screening for latent TB is recommended before starting bDMARDS or tsDMARDs.	**100%**
	1.2. Screening should be considered in patients treated with glucocorticoids depending on the doses, duration of treatment, and risk factors: *1.2.1.* Screening should be considered particularly in those patients likely to receive at least 20 mg of prednisolone (or equivalent)/day for more than 2-4 weeks; the treating physician should decide whether their patients are at risk and would benefit from a screening for latent TB	**86%**
	*1.2.2.* For patients who have received doses of prednisolone ranging from 7.5-20 mg (or equivalent)/day for less than three months, the treating physician should decide whether their patients are at risk and would benefit from a screening for latent TB.	**89%**
	1.3. Screening is conditionally recommended before initiating csDMARDs based on the clinical assessment of the treating physician and individual risk factors.	**100%**
	1.4. Physicians are requested not to start treatment before getting the screening results.	**83%**
**Statement 2:** Methods and periodicity of screening	2- Screening for latent TB should typically include a chest X-ray and Interferon-gamma release assay (IGRA)	**100%**
	2.1. In addition to chest X-ray, IGRA (TB QuantiFERON gold test) should be preferred over Tuberculin Skin Test (TST) for TB screening.	**100%**
	2.2. There is no consensus nor robust data for how often periodic prescreening should be performed. The IGRA test might be assessed periodically, depending on the clinical judgment of the physician and any change in the patient’s clinical condition.	**89%**
	2.3. IGRA TB QuantiFERON test is performed for the screening of latent TB in the pediatric population. However, limitations are noted below some group ages (3 or 5 years, depending on references). Referral to ID specialists is warranted.	**78%**
**Statement 3:** Strategies for a positive IGRA test	3- In case of a positive IGRA test, the patient should be assessed for latent or active TB, as indicated in Table **2** and the algorithm in Fig. (**2**). 3.1. If latent or active TB is diagnosed, then the patient should be referred to an ID specialist or pulmonologist for further assessment and should start treatment with particular attention to the interaction with medications used to treat AIIRD. 3.2. It is not recommended that TB be managed by rheumatologists; patients should be referred to specialists. 3.3. If latent TB is diagnosed, treatment for TB should be initiated, and biological treatment can be started after four weeks (Treatment regimens in Table **3**).	**94%**
**Statement 4:** **Hepatitis B**	**Hepatitis B** 4- All patients considered for treatment with bDMARDs, tsDMARDs, immunosuppressants, and glucocorticoids (20 mg of prednisolone (or equivalent)/day for more than 2-4 weeks) should be screened for HBV. 4.1. The risk of HBV reactivation should be assessed before starting csDMARDs, according to the treating physician’s judgment and on an individual, case-by-case basis. 4.2. Typical screening for hepatitis B virus (HBV) status includes hepatitis B surface antigen (HBsAg), hepatitis B core antibody (anti-Hbcore), and hepatitis B surface antibody immunity (anti-HBs). 4.3. HBV status should be determined before initiating the treatment for AIIRD and deciding whether vaccination is needed. HBV status reflects that the patient may be: - unexposed - vaccinated - carrier (HBsAg-positive) - resolved (anti-HBcore-positive and HBsAg-negative). 4.4. Table **4** summarizes the suggested procedures based on HBV tests and status. 4.5. The risk of HBV reactivation should be assessed on a case-by-case basis for patients likely to receive at least 20 mg of prednisolone (or equivalent)/day for more than 2-4 weeks [15]. 4.6. Antiviral therapy should start ideally before or at least simultaneously with the treatment for AIIRD and continue for at least 6-12 months after discontinuing antirheumatic treatment. 4.7. Periodic testing (adult population): There is a lack of data on when to re-screen for HBV reactivation. Periodic testing should be performed based on individual evaluations, considering risk factors and costs. Referral to hepatologists is also recommended. 4.8. Periodic testing (pediatric population): If the screening for HBV is performed before initiating treatment with bDMARDs, periodic testing will not be repeated unless the treating physician decides otherwise (*e.g*., after blood transfusion, if liver enzymes are abnormal, or if other risk factors are identified).	**85%**
**Statement 5:** **Hepatitis C**	**Hepatitis C** 5- Screening for chronic hepatitis C (HCV) should be considered before initiating csDMARDs, bDMARDs, tsDMARDs, immunosuppressants, and glucocorticoids (according to dose and duration). Screening is recommended for patients with elevated alanine aminotransferase or those with known risk factors. 5.1. Screening for HCV is mandatory before starting bDMARDs and tsDMARDs. 5.2. Screening for HCV should be done before starting prednisolone at doses of at least 20 mg of prednisolone (or equivalent)/day for more than 2-4 weeks. 5.3. Screening for HCV should be done on a case-by-case basis before starting csDMARDs. 5.4. Screening for HCV includes anti-HCV antibodies; if these are present, the patient should be referred to a hepatologist or ID specialist for further testing/evaluation and treatment.	**89%**
**Statement 6: HIV**	**HIV** 6- Screening for HIV is considered based on high-risk situations assessed by the treating physician. 6.1. Screening for HIV *via* HIV-Ag (1+2) should be done once before treatment with bDMARDs and tsDMARDs (conditional); if the test is positive, the patient should be referred to an ID specialist. 6.2. No screening is recommended for pediatric populations.	**94%**
**Statement 7:** **Varicella Zoster Virus**	**Varicella Zoster Virus** 7- All patients who are non-immune to Varicella Zoster virus (VZV) and are starting csDMARDs, bDMARDs, tsDMARDs, immunosuppressants, and/or glucocorticoids should be informed about post-exposure prophylaxis following contact with VZV. 7.1. No serological screening for VZV immunity is recommended for patients before starting csDMARDs, bDMARDs, tsDMARDs, immunosuppressants, and/or glucocorticoids. 7.2. Vaccination should be offered to all patients before initiating tsDMARDs and bDMARDs to prevent the disease or its complications. 7.3. Vaccination is recommended with the non-live recombinant subunit adjuvant zoster vaccine (cf. section V.4.2). 7.4. Any physician, including rheumatologists or ID specialists, can recommend vaccination and administer the vaccine.	**94%**
**Statement 8: *Pneumocystis*** ***jirovecii***	***Pneumocystis jirovecii*** - Prophylaxis against *Pneumocystis jirovecii* pneumonia (PCP) should be considered in patients with AIIRD in whom high doses of glucocorticoids (at least 20 mg of prednisolone (or equivalent)/day for more than 2-4 weeks) are used, especially in combination with immunosuppressants and depending on the risk-benefit ratio and according to the physician’s judgment and clinical condition. 8.1. The prophylaxis scheme should be decided based on a shared discussion between the rheumatologist and ID specialist, depending on the patient’s condition and associated comorbidities. 8.2. The main prophylaxis scheme is trimethoprim/sulfamethoxazole (TMP-SMX) 480 mg/day (single-strength) or 960 mg three times a week (Table **5**; Supplementary Table **3**). 8.3. Other alternative regimens, equally efficacious and with fewer adverse effects, can also be suggested (Table **5**; Supplementary Tables **2** and **3**). 8.4. In case of allergy or reported side effects to TMP-SMX, alternative prophylactic medications can be suggested depending on the availability and accessibility in the different institutions. The first suggested option is dapsone. Other alternatives include atovaquone and nebulized pentamidine. 8.5. Atovaquone should be considered the preferred option over TMP-SMX for patients with Systemic Lupus Erythematosus (SLE) who are at a higher risk of experiencing allergic reactions or lupus flares. The use of TMP-SMX in such cases can lead to serious consequences, including severe lupus flares and allergic reactions. 8.6. Atovaquone should be considered the preferred option over TMP-SMX, specifically in Caucasian patients, individuals with lymphopenia, and those who test positive for anti-SSA antibodies. This subgroup of patients faces a higher risk of experiencing mixed reactions when TMP-SMX is administered.	**94%**

**Abbreviations:** AIIRD: Autoimmune inflammatory rheumatic diseases; bDMARDs: Biological disease-modifying antirheumatic drugs; csDMARDs: Conventional disease-modifying antirheumatic drugs; HBV: Hepatitis B virus; HBsAg: Hepatitis B surface antigen; Anti-Hbcore: Hepatitis B core antibody; anti-HBs: Hepatitis B surface antibody immunity; HCV: Hepatitis-C virus; HIV: human immunodeficiency virus; HPV: Human papilloma virus; ID: ID: Infectious diseases; IGRA: Interferon-gamma release assay; PCP: *Pneumocystis jirovecii* pneumonia; SLE: Systemic Lupus Erythematosus; TB: Tuberculosis; TMP-SMX: Trimethoprim/sulfamethoxazole; tsDMARDs: Targeted synthetic disease-modifying antirheumatic drugs; TST: Tuberculin Skin Test; VZV: Varicella zoster virus [7].

**Table 2:** TB status in IGRA-positive patients.

-	**Latent TB (Patient Exposed to TB)**	**Active TB**
Chest X-Ray	Normal	Abnormal
Symptoms	None	Yes
Contagious	No	Yes

**Abbreviations:** IGRA: Interferon-gamma release assay; TB: Tuberculosis.

**Table 3:** Latent tuberculosis treatment regimens.

**Regimen**	**Dosing**	**Duration**
Isoniazid^1^	Daily dose: 5 mg/Kg Max dose: 300 mg daily	6-9 months
Combination of rifampicin/isoniazid	Daily dose: 10 mg/Kg- 5 mg/Kg Max dose: 600 /300 mg daily	3-4 months
Rifampicin	Daily dose: 10 mg/Kg Max dose: 600 mg daily	4 months

**Note: ^1^**The global and UAE monoresistance rate of isoniazid is approximately 10%. This resistance rate should be taken into consideration when initiating treatment.

**Table 4:** Suggested procedures based on HBV tests and status.

**HBV-Status**	**HbsAg**	**Anti-Hbcore**	**HBV-DNA**	**Anti-HBs**	**Management Strategy**
Carriers	+				Refer to a hepatologist or infectious disease specialist for antiviral prophylactic treatment
Resolved	-	+			Monitor and refer to hepatologist or infectious disease specialist for antiviral prophylactic treatment when necessary
Resolved with reactivation risk	-	+	-		Monitor and refer to hepatologist or infectious disease specialist for antiviral prophylactic treatment when necessary
Reactivation of a resolved-HBV	-	+	+		Monitor and refer to hepatologist or infectious disease specialist for antiviral prophylactic treatment when necessary
Prior vaccination	-	-		+	Usual follow-up
No immunity nor previous infection	-	-		-	Recommend vaccination

**Abbreviations:** HBV: Hepatitis B virus; HBsAg: Hepatitis B surface antigen; Anti-Hbcore: Hepatitis B core antibody; anti-HBs: Hepatitis B surface antibody immunity.

**Table 5:** Regimens for *Pneumocystis pneumonia* prophylaxis in adults and adolescents.

**Drug**	Oral Dose*	**Selected Adverse Reactions**
**Preferred Regimen**		
Trimethoprim-sulfamethoxazole (cotrimoxazole)	1 DS tablet daily OR 1 SS tablet daily	Fever, rash, neutropenia, gastrointestinal upset, transaminase elevation
**Alternative Regimen**		
Trimethoprim-sulfamethoxazole (cotrimoxazole)	1 DS tablet three times per week	Fever, rash, neutropenia, gastrointestinal upset, transaminase elevation
Dapsone	50 mg twice daily OR 100 mg daily	Fever, rash, gastrointestinal upset, methemoglobinemia, hemolytic anemia (check for G6PD deficiency)
Atovaquone suspension	1500 mg orally once daily given with food	Gastrointestinal distress, rash
Combination of: Dapsone Pyrimethamine Leucovorin	50 mg daily plus 50 mg weekly plus 25 mg weekly	Fever, rash, gastrointestinal upset, methemoglobinemia, hemolytic anemia (check for G6PD deficiency) Folate deficiency, gastrointestinal upset, rash Rash, thrombocytosis, wheezing, anaphylactoid reactions
Aerosolized pentamidine	300 mg monthly (*via* Respirgard II nebulizer)	Cough, wheezing, extrapulmonary pneumocystosis

**Note:** *The doses recommended in the table are intended for patients with normal renal function; the doses of some of these agents must be adjusted in patients with renal insufficiency.

**Abbreviations:** DS: double-strength oral tablet, 160 mg trimethoprim with 800 mg sulfamethoxazole; SS: single-strength oral tablet, 80 mg trimethoprim with 400 mg sulfamethoxazole; G6PD: glucose-6-phosphate dehydrogenase; PCP: *Pneumocystis jirovecii* pneumonia; HIV: human immunodeficiency virus; IgG: immunoglobulin G; TMP-SMX: trimethoprim/sulfamethoxazole.

**Table 6:** The Emirati consensus recommendations for vaccination in adults with autoimmune inflammatory rheumatic diseases.

**-**	**Statements**	**Level of Agreement**
**V.0. General statement**	V.0. Vaccine recommendations might change based on updated Centers for Disease Control and Prevention (CDC)/ Advisory Committee on Immunization Practices (ACIP) guidelines.	**100%**
**V.1. Influenza vaccination**	V.1.1. The influenza vaccine is strongly recommended yearly for patients with AIIRD undergoing immunosuppressive therapy. V.1.2. Rheumatologists should review vaccination history annually. Table **7** presents a summarized guideline for the administration of the influenza vaccine and other non-live attenuated vaccines.	**100%**
**V.2. Pneumococcal vaccination**	V.2.1. Physicians should check the CDC guidelines for pneumococcal vaccination due to the rapid changes in this area. V.2.3. The most recommended regimen for the pneumococcal vaccination would include the administration of the PCV13 followed by the PPSV23 ≥5 years since the last vaccination dose. Other regimens are also available as recommended by the CDC. V.2.4. PCV20 recommendations: • V.2.4.1. For those who have not previously received any pneumococcal vaccine, the CDC recommends the administration of one dose of PCV15 or PCV20: - If PCV15 is used, it should be followed by a dose of PPSV23 at least one year later. The minimum interval is eight weeks and can be considered in adults with an immunocompromising condition, cochlear implant, or cerebrospinal fluid leak. - If PCV20 is used, a dose of PPSV23 is not indicated. • V.2.4.2. For those who have only received PPSV23, the recommendation for one dose of PCV15 or PCV20 is as follows: - The PCV15 or PCV20 dose should be administered at least one year after the most recent PPSV23 vaccination. - Regardless of whether PCV15 or PCV20 is given, an additional dose of PPSV23 is not recommended in those persons since they have already received it. • V.2.4.3. For those who have only received PCV13, the CDC recommends the administration of either one dose of PCV20 at least one year after PCV13 and one dose of PPSV23; the minimum interval between the PCV13 and PPSV23 doses will vary depending on their specific risk factor. V.2.5. In patients with an immunocompromising condition (at least eight weeks after PCV13), the CDC recommends the administration of up to two additional doses of PPSV23 in their lifetime. The second dose of PPSV23 should be administered at least five years after the first dose of PPSV23. If a patient is 65 years or older at the time of the second dose, then a third dose is not indicated. For patients who have received PCV13 and one dose of PPSV23, the CDC recommends the administration of either one dose of PCV20 at least five years after the last pneumococcal vaccine or a second dose of PPSV23 at least eight weeks after PCV13 and five years after PPSV23 if they have an immunocompromising condition.	**94%**
**V.3. Human papillomavirus (HPV) vaccination**	V.3.1. For patients with AIIRD aged between 26 and 45 and receiving immunosuppressive medication and not previously vaccinated, vaccination against human papillomavirus (HPV) is conditionally recommended (optional) based on a shared clinical decision between the physician and the patient. V.3.2. Clinicians are advised to follow the national vaccination guidelines in the UAE for adult and pediatric populations.	**100%**
**V.4. Varicella-zoster vaccination (VZV)**	V.4.1. The recombinant varicella-zoster vaccine (VZV) is strongly recommended for patients with AIIRD aged 18 years and above receiving immunosuppressive medication.V.4.2. The type of vaccine and suggested regimen are summarized in Table **8**. V.4.3. It is unnecessary to stop immunosuppressive medication before administering the vaccine. V.4.4. Antipyretic medication (*e.g*., paracetamol/acetaminophen) is not recommended before vaccination.	**89%**
**V.5. Tetanus** **vaccination**	V.5.1. For patients with AIIRD who are taking immunosuppressive medication, it is generally considered safe to administer a diphtheria/tetanus vaccine booster, particularly for all adults who sustain dirty wounds and are either unvaccinated or have not received a primary series of tetanus toxoid-containing vaccines. In addition to the vaccine, it is also recommended to administer tetanus immunoglobulin (TIG) for prophylaxis. V.5.2. The recommended dose of TIG for prophylaxis is 250 IU, administered intramuscularly.	**89%**
**V.6. COVID-19 vaccination and prophylaxis**	V.6.1. Patients with AIIRD should receive a COVID-19 vaccine unless contraindicated. V.6.2. The mRNA vaccine is recommended in AIIRD patients who are not yet vaccinated. V.6.3. For AIIRD patients receiving any immunosuppressive or immunomodulatory therapy who have already received two doses of mRNA vaccine, an additional dose is recommended ≥28 days after the end of the vaccine series..6.4. it is not recommended for healthcare providers to routinely request laboratory tests (*e.g*., antibody tests for IgM and/or IgG against spike or nucleocapsid proteins) to assess immunity to COVID-19 post-vaccination or the need for vaccination in an as-yet-unvaccinated person. V.6.5. AIIRD patients not receiving immunomodulatory treatments should take the first dose of the COVID-19 vaccine before initiating immunomodulatory therapy when feasible. V.6.6. AIIRD patients at high risk for poor outcomes related to COVID-19 should receive monoclonal antibody therapy preventively (pre-exposure prophylaxis). V.6.7. AIIRD patients can be offered long-acting monoclonal antibodies with confirmed efficacy for pre-exposure prophylaxis against COVID-19 in case the circulating variants of concerns are still neutralized. This recommendation applies even to patients who have been vaccinated against COVID-19, are not currently infected with SARS-CoV-2, have not had known recent exposure to an infected individual, and those who have moderate to severe immune compromise, such as patients with AIIRD or undergoing treatments and who may not develop a sufficient immune response to COVID-19 vaccination. V.6.8. AZD3152 (Evusheld 2.0) is an investigational, long-acting antibody that has been developed to have broad neutralizing activity across SARS-CoV-2 strains. Current evidence has shown that compared with Evusheld^1^, ASD3152 (Evusheld 2.0) exhibits higher efficacy and a more prolonged activity. V.6.9. Refer to national guidelines for detailed recommendations regarding prophylaxis and treatment of COVID-19.	**83%**

**Abbreviations:** ACIP: Advisory Committee on Immunization Practices; AIIRD: Autoimmune inflammatory rheumatic diseases; CDC: Centers for Disease Control and Prevention; HPV: Human papilloma virus; PCV13: 13-valent pneumococcal conjugate vaccine; PCV15: 15-valent pneumococcal conjugate vaccine; PCV20: 20-valent pneumococcal conjugate vaccine; PPSV23: 23-valent pneumococcal polysaccharide vaccine; TIG: Tetanus immunoglobulin; VZV: Varicella zoster virus (Adapted from: [6, 7, 15, 31, 32]).

**Table 7:** Administration of Influenza vaccine and other non-live attenuated vaccinations based on the patients’ treatment.

**Treatments**	**Influenza Vaccine**	**Other Non-live Attenuated Vaccinations**
Methotrexate^1^	Continue methotrexate	Continue methotrexate
Rituximab^2^	Continue rituximab	Time vaccination for when the next rituximab dose is due, and then hold rituximab for at least 2 weeks after vaccination
Immunosuppressive medications other than methotrexate and rituximab	Continue immunosuppressive medication	Continue immunosuppressive medication
Prednisolone: - Prednisone ≤ 10 mg daily - Prednisone > 10 mg and <20 mg daily - Prednisone ≥ 20 mg daily	Give Give Give	Give Give Defer

**Note:** ^1^ **Methotrexate:** A mismatch between the 2022 American guidelines [7] and the 2019 EULAR guidelines [6, 31] regarding the discontinuation of MTX with influenza vaccination is noted. Hence, the ACR guidelines state that temporary discontinuation of MTX was shown to improve the immunogenicity of seasonal influenza vaccination in patients with RA, with the best results when MTX was suspended for 2 weeks before and 2 weeks after vaccination. However, it is currently not recommended to stop MTX before or after

^2^ **Rituximab:** For patients treated with rituximab and abatacept, a second boosting dose of vaccine is recommended to improve immunogenicity and lead to levels of seroprotection comparable to healthy controls [6].

**Abbreviations:** ACR: American College of Rheumatology; EULAR: EULAR: European Alliance of Associations for Rheumatology; MTX: Methotrexate; RA: Rheumatoid Arthritis.

**Table 8:** Recommendation for VZV vaccination in patients with AIIRD according to the 2019 EULAR recommendations.

**Type of Vaccine**	**Suggested Regimen**	**Comments**
Non-live recombinant subunit adjuvant zoster vaccine (Shingrix™):	Two intramuscular doses provided 1 to 6 months apart [14].	Safety and efficacy of the subunit zoster vaccine have not yet been investigated in AIIRD patients. Nevertheless, based on the fact that Shingrix is a non-live vaccine, it may replace the live-attenuated vaccine in patients with AIIRD.

**Abbreviations:** AIIRD: Autoimmune Inflammatory Rheumatic Diseases, EULAR: European Alliance of Associations for Rheumatology, VZV: Varicella Zoster Virus.

**Note:** [31].

## Data Availability

The datasets generated during the current study are available from the corresponding author upon request.

## Footnotes

1 The SUPERNOVA Phase I/III trial will assess the safety and neutralizing activity of AZD3152 in preventing symptomatic COVID-19 in adults and adolescents aged 12 years and older with conditions that compromise immune function

## LIST OF ABBREVIATIONS

ACIP: Advisory Committee on Immunization Practices
ACR: American College of Rheumatology
AIIRD: Autoimmune Inflammatory Rheumatic Diseases
Anti-Hbcore: Hepatitis B Core Antibody
anti-HBs: Hepatitis B Surface Antibody Immunity
bDMARDs: Biological Disease-Modifying Antirheumatic Drugs
CDC: Centers for Disease Control and Prevention
csDMARDs: Conventional Disease-Modifying Antirheumatic Drugs
DS: Double-Strength Oral Tablet, 160 mg Trimethoprim with 800 mg Sulfamethoxazole
EULAR: European Alliance of Associations for Rheumatology
G6PD: Glucose-6-Phosphate Dehydrogenase
HBsAg: Hepatitis B Surface Antigen
HBV: Hepatitis B Virus
HCV: Hepatitis C Virus
HIV: Human Immunodeficiency Virus
HPV: Human Papilloma Virus
ID: Infectious Diseases
IgG: Immunoglobulin G
IGRA: Interferon-Gamma Release Assay
MTX: Methotrexate
PCP: Pneumocystis Jirovecii Pneumonia
PCV13: 13-Valent Pneumococcal Conjugate Vaccine
PCV15: 15-Valent Pneumococcal Conjugate Vaccine
PCV20: 20-Valent Pneumococcal Conjugate Vaccine
PPSV23: 23-Valent Pneumococcal Polysaccharide Vaccine
RA: Rheumatoid Arthritis
SLE: Systemic Lupus Erythematosus
SS: Single-Strength Oral Tablet, 80 mg Trimethoprim with 400 mg Sulfamethoxazole
TB: Tuberculosis
TIG: Tetanus Immunoglobulin
TMP-SMX: Trimethoprim-Sulfamethoxazole
tsDMARDs: Targeted Synthetic Disease-Modifying Antirheumatic Drugs
TST: Tuberculin Skin Test
VZV: Varicella Zoster Virus

## SUPPLEMENTARY MATERIAL

Supplementary Table 1: Available medications for AIIRD in the UAE. Supplementary Table 2: Treatment options for patients with latent tuberculosis Supplementary Table 3: PCP Prophylaxis with trimethoprim-sulfamethoxazole (TMP-SMX) in patients with AIIRD (level of evidence) Supplementary Table 4: Available vaccines in the UAE. Supplementary Table 5: Immunosuppressive medication management at the time of live attenuated virus vaccine administration Supplementary Table 6: Guidance Related to the Use and Timing of Vaccine Dosing and Immunomodulatory Therapy in Relation to COVID-19 Vaccination in patients with AIIRD. Supplementary Table 7: Dosage of EVUSHELD for prophylaxis. Supplementary material is available on the publisher’s website along with the published article.
