# Supplementary material for: Infection Screening and Vaccination of Adult and Pediatric Patients with Autoimmune Inflammatory Rheumatic Diseases: An Emirati Delphi Consensus
Source: Curr Rheumatol Rev. 2025 Jan 21;21(5):545–61. doi: 10.2174/0115733971368196250117105119 (PMC12824857; doi:10.2174/0115733971368196250117105119)
Supplement: Supplementary file 1 [file CRR-21-5-545_SD1.pdf]

Supplementary Material

Infection Screening and Vaccination of Adult and Pediatric Patients with Autoimmune Inflammatory Rheumatic Diseases: An Emirati Delphi Consensus

Ahlam Almarzooqi[1,*], Jehad Abdalla[2], Mohamed Sharif Elsadeg[3], Noura Zamani[4], Amel Abdel Gadir Ginawi[5], Zaid Alrawi[6], Rajaie Namas[7], Afra Aldhaheri[8], Ahmed Zayat[9], Faisal Elbadawi[4], Layla ALDabal[10], Najla Aljaberi[11], Shazia Abdullah[12], Suad Hannawi[13], Khalid A. Alnaqbi[14], Fatima Al Dhaheri[15], Beena Hameed[16] and Jamal Al-Saleh[4]

[1]Department of Rheumatology, Al Qassimi Hospital, Emirates Health Services, Sharjah, United Arab Emirates; [2]Department of Infectious Diseases, Sheikh Khalifa Medical City (SKMC), Abu Dhabi, United Arab Emirates; [3]Pediatric Rheumatology, Al Jalila Children Hospital, Dubai, United Arab Emirates; [4]Department of Rheumatology, Dubai Hospital, Dubai Academic Health Corporation, Dubai, United Arab Emirates; [5]Department of Rheumatology, Mediclinic City Hospital, Dubai, United Arab Emirates; [6]Department of Rheumatology, Clemenceau Medical Center Hospital, Dubai, United Arab Emirates; [7]Department of Rheumatology, Medical Specialties Institute, Cleveland Clinic Abu Dhabi, Abu Dhabi, United Arab Emirates; [8]Internal Medicine, Tawam Hospital, Al Ain, United Arab Emirates; [9]Department of Rheumatology, University Hospital Sharjah, University of Sharjah, Sharjah, United Arab Emirates; [10]Rashid Hospital, Dubai Academic Health Corporate, Infectious Disease, Dubai, United Arab Emirates; [11]Department of Pediatrics, College of Medicine & Health Sciences, United Arab Emirates University, Al Ain, United Arab Emirates; [12]Department of Pediatrics, Sheikh Khalifa Medical City, Abu Dhabi, United Arab Emirates; [13]Rheumatology, AlKuwait-Dubai hospital (ALBaraha), Emirates Health Services (EHS), Ministry of Health and Prevention (MOHAP), Dubai, United Arab Emirates; [14]Rheumatology, Tawam Hospital, College of Medicine and Health Sciences, UAE University, Al Ain, United Arab Emirates; [15]College of Medicine and Health Sciences, Infectious Disease, UAEU, Al Ain, United Arab Emirates; [16]Internal Medicine, King's College Hospital Dubai, Dubai, United Arab Emirates

1875-6360/25 **© 2025 Bentham Science Publishers**

Supplementary Table 1: Available medications for AIIRD in the UAE.

Immunosuppressive	Non-immune suppressive
Glucocorticoids (Prednisone, Methylprednisolone, Dexamethasone, Hydrocortisone)<br>csDMARDs (Methotrexate, Leflunomide, Azathioprine, Mycophenolate mofetil/ mycophenolic acid)<br>Calcineurin inhibitors: cyclosporine, tacrolimus, voclosporin)<br>Cyclophosphamide<br>bDMARDs<br>TNF inhibitors (etanercept, adalimumab, certolizumab, golimumab, infliximab)<br>IL-6R inhibitors (tocilizumab)<br>IL-17 inhibitors (secukinumab, ixekizumab)<br>IL-12/23 inhibitors (ustekinumab)<br>IL-23 inhibitors (guselkumab, Risankizumab)<br>IL-1 inhibitors (anakinra, canakinumab)<br>T cell co-stimulation inhibitor (CTLA4-Ig/ abatacept)<br>B cell–depleting agents (rituximab)<br>BlyS/BAFF inhibitors (belimumab)<br>Interferon-α receptor inhibitor (anifrolumab)<br>tsDMARDs -JAK inhibitors (tofacitinib, baricitinib, upadacitinib,)	Hydroxychloroquine<br>Sulfasalazine<br>Colchicine<br>Apremilast<br>Denosumab<br>IVIG

Supplementary Table 2: Treatment options for patients with latent tuberculosis [1-3].

Medication	Treatment duration	Interactions	Main side effects (>10%) [2, 3]
Isoniazid	6-12 months	Monitor liver function tests (LFTs), especially if cotreatment with hepatotoxic drugs like methotrexate and leflunomide	- Mild increase in LFTs (10-20%)<br>- Peripheral neuropathy (dose-related incidence, 10-20% incidence with 10 mg/kg/d)<br>- Loss of appetite<br>- Nausea<br>- Vomiting<br>- Stomach pain<br>- Weakness
Rifampicin/Isoniazid	3-4 months	Isoniazid: Monitor liver function tests (LFTs), especially if cotreatment with hepatotoxic drugs like methotrexate and leflunomide<br><br>Rifampicin: Consider check-	

		ing the pharmacokinetics of JAK-inhibitors and glucocorticoids	
Rifampicin for 4 months and once-weekly therapy of isoniazid plus rifapentine		Rifampicin: Consider checking the pharmacokinetics of JAK-inhibitors and glucocorticoids	Rifapentine: hyperuricemia

Supplementary Table 3: PCP Prophylaxis with trimethoprim-sulfamethoxazole (TMP-SMX) in patients with AIIRD (level of evidence) (Adapted from [4]).

Study	Treatment	Treatment scheme	Suggested prophylaxis regimen for PCP	Outcomes of prophylaxis & alternative dosing	RoB
Park et al. 2018 [5]	GC	≥30 mg/day for ≥4 weeks	TMP-SMX 80/400 mg/day* or 160-800 mg** three times a week	Reduced PCP incidence	8
Honda et al. 2019 [6]	GC	≥50 mg/day	TMP-SMX 80/400 mg/day* or less	Reduced PCP incidence	7
Park et al. 2019 [7]	GC	Two groups: 1- <15 mg/day (low dose) for ≥4 weeks (prolonged treatment) 2- ≥15 mg/day and <30 mg/day (medium dose) for ≥4 weeks (prolonged treatment)	TMP-SMX 80/400 mg/day*	Reduced PCP incidence in high risk group (GC-pulse or lymphopenia)	7
Ogawa et al. 2005 [8]	GC	≥30 mg/day for 2 weeks	TMP-SMX 80/400 mg/day* or 3 tablets three times a week	Effective in high-risk patients (risk prediction model)	7
Vananuvat et al. 2011 [9]	GC	≥20 mg/day for >2 weeks	TMP-SMX 480 mg/day (single-strength) or 960 mg three times a week	Reduced PCP incidence	6

Utsunomiya et al. 2017^ [10]	GC	Prednisolone >0.6 mg/kg/day	SS (n=58), HS (n=59), ES (n=55)	-0%PCP for all groups (week 24)<br>-HS and ES (vs SS): lower AE and lower discontinuation rates	High
Utsunomiya et al. 2020^ [11]	GC	Prednisolone >0.6 mg/kg/day	SS (n=58), HS (n=59), ES (n=55)	-0%PCP for all groups (week 52)<br><br>-HS and ES (vs SS): lower AE and lower discontinuation rates	Some concerns
Harada et al. 2021 [12]	Antirheumatic drugs	-	SS or 960/thrice weekly (n=145) Vs dose-reduction (n=75)^.	-PCP 0% for all groups<br>-SS: more AEs	8
Takenaka et al. 2013 [13]	Antirheumatic drugs	-	Routine group (n=28) Vs. Escalation group (n=13)	Escalation group: higher retention	6
Suyama et al. 2016 [14]	Immunosuppressants	-	SS (n=31) Vs graded administration (n=28)	Graded administration had less AE	5

Abbreviations: AE: adverse events; ES: escalated strength group (started with 8/40 mg daily, increasing incrementally to 40/200 mg daily); GC: Glucocorticoids; HS: half strength (40/200 mg daily); PCP: *Pneumocystis jirovecii* pneumonia; RoB: Risk of bias assessed by Newcastle-Ottawa scale (score 0-9; a study with score from 7-9, has high quality, 4-6, high risk, and 0-3 very high risk of bias) for all studies except RCTs in which Cochrane risk of bias tool (score for risk of bias: low, high, some concerns) was used; TMP-SMX: Trimethoprim-sulfamethoxazole;
*Single strength containing 80 mg of TMP and 400 mg of SMX; **Double strength containing 160 mg of TMP and 800 mg of SMX; ^ RCTs.

Supplementary Table 4: Available vaccines in the UAE.

PHX CODE	Vaccine's name	Dosage
PHX03454	CHOLERA, INACTIVATED. ORAL VACCINE	single dose vial
PHX02087	DIPHTHERIA / TETANUS / ACELLULAR PERTUSSIS / HAEMOPHILUS B / HEPATITIS B/INJECTABLE POLIO (DTaP/Hib/HepB,IPV)(Hexa)	single dose injection
PHX03239	DIPHTHERIA / TETANUS / ACELLULAR PERTUSSIS / HAEMOPHILUS INFLUENZAE B / INJECTABLE POLIO (DTaP,Hib,IPV), (Tetra-IPV)	single dose injection

PHX00607	HEMOPHILUS INFLUENZA TYPE B CONJUGATE	single dose injection
PHX02608	HEPATITIS A INACTIVATED, ADULT (HepA Ad),	single dose vial
PHX03429	HEPATITIS A INACTIVATED, PEDIATRIC (HepA pd)	Single Dose Vial
PHX00621	HEPATITIS B VACCINE (Recombinant, Inactivated) (HepB ped.)	10 mcg (pediatric) injection (single dose)
PHX00620	HEPATITIS B VACCINE (Recombinant, Inactivated) (HepB adult)	20 mcg (adult) single dose injection
PHX02595	HUMAN PAPILLOMAVIRUS BIVALENT (HPV2)	single dose injection
PHX02596	HUMAN PAPILLOMAVIRUS QUADRIVALENT (HPV4)	single dose injection
PHX03453	INFLUENZA VACCINE (SEASONAL), INACTIVATED QUADRIVALENT (IIV4)	single dose prefilled syringe
PHX00667	INFLUENZA VACCINE (SEASONAL), INACTIVATED TRIVALENT (IIV3)	single dose injection
PHX03460	INFLUENZA VACCINE (SEASONAL), INACTIVATED TRIVALENT (IIV3), pediatric	single dose injection
PHX01312	MEASLES / MUMPS / RUBELLA VACCINE (Live Attenuated) (MMR)	single dose injection
PHX02441	MENINGOCOCCAL ACYW135 CONJUGATED, (MCV4)	single dose injection vial
PHX03404	POLIO VIRUS (live attenuated type 1 and 3) / BIVALENT (bOPV)	oral , 20 doses / vial
PHX02607	ROTAVIRUS MONOVALENT (RV1), ORAL	single does prefilled syringe
PHX02444	ROTAVIRUS PENTAVALENT (RV5), oral	single dose tube
PHX01199	TETANUS TOXOID ADSORBED (TT)	single dose injection
PHX01244	TUBERCULIN TEST	(PPD 5),5 TU/ Test (vial)
PHX01245	TYPHOID VACCINE (purified Vi polysaccharide of S. Typhi bacteria)	single dose injection
PHX01913	Vaccine, BACILLUS CALMETTE-GUERIN BCG	10/20 doses / unit, Plus Diluent 1 mL / unit
PHX01915	VACCINE, DIPHTHERIA, TETANUS, ACELLULAR PERTUSSIS, (DTaP), for below 7 years	single dose injection
PHX03539	VACCINE, DIPHTHERIA, TETANUS, ACELLULAR PERTUSSIS, INACTIVATED POLIO (DTaP, IPV), for below 7 years	single dose injection
PHX04092	VACCINE, Influenza Inactivated High-Dose quadrivalent (seasonal), (IIV4-HD)	single dose injection
PHX03540	VACCINE, HUMAN PAPILLOMAVIRUS, 9 VALENT (HPV9)	single dose injection
PHX02301	VACCINE, PNEUMOCOCCAL CONJUGATE, 13 VALENT (PCV13)	single dose injection
PHX04093	VACCINE, Pneumococcal conjugate, 20 valent, (PCV20), single dose	single dose injection
PHX01530	VACCINE, PNEUMOCOCCAL POLYSACCHARIDE, 23 valent, (PPSV23)	single dose injection

PHX01061	VACCINE, RABIES, VERO CELLS (Rab-Vero)	single dose injection
PHX04038	VACCINE, RECOMBINANT HERPES ZOSTER	0.5 mL single dose injection
PHX02440	VACCINE, TETANUS, REDUCED DIPHTHERIA, REDUCED ACELLULAR PERTUSSIS, (TdaP), for 7 years and above	single dose injection
PHX02330	VACCINE,TETANUS, REDUCED DIPHTHERIA (Td), for 7 years and above	single dose injection
PHX01257	VARICELLA VACCINE (Live Attenuated) (Var)	single dose injection
PHX01920	YELLOW FEVER (Live Attenuated) (YF)	single dose injection

Supplementary Table 5: Immunosuppressive medication management at the time of live attenuated virus vaccine administration [15].

Immunosuppressive medication	*Hold before live-attenuated virus vaccine administration*	*Hold after live-attenuated virus vaccine administration*
Glucocorticoids	4 weeks	4 weeks
Methotrexate, azathioprine	4 weeks	4 weeks
Leflunomide, mycophenolate mofetil, calcineurin inhibitors, oral cyclophosphamide	4 weeks	4 weeks
JAK inhibitors	1 week	4 weeks
TNF, IL17, IL12/23, IL23, BAFF/BLyS inhibitors	1 dosing interval	4 weeks
IL6 pathway inhibitors	1 dosing interval	4 weeks
IL1 inhibitors<br>Anakinra<br>Rilonacept<br>Canakinumab	1 dosing interval<br>1 dosing interval<br>1 dosing interval	4 weeks<br>4 weeks<br>4 weeks
Abatacept	1 dosing interval	4 weeks
Anifrolumab	1 dosing interval	4 weeks
Cyclophosphamide IV	1 dosing interval	4 weeks
Rituximab	6 months	4 weeks
IVIG e<br>300-400 mg/kg<br>1 gm/kg<br>2 gm/kg	8 months<br>10 months<br>11 months	4 weeks<br>4 weeks<br>4 weeks

Supplementary Table 6: Guidance Related to the Use and Timing of Vaccine Dosing and Immunomodulatory Therapy in Relation to COVID-19 Vaccination in patients with AIIRD.

Medication	Timing Considerations for Immunomodulatory Therapy and Vaccination (applies to both primary vaccination and supplemental [booster] dosing)
Abatacept IV	Time vaccination so that it occurs one week prior to the next dose of IV abatacept
Abatacept SC	Hold for one to two weeks (as disease activity allows) after each

	COVID vaccine dose
Acetaminophen, NSAIDs	Assuming that disease is stable, hold for 24 hours prior to vaccination. No restrictions on use post vaccination once symptoms develop.
Belimumab SC	Hold for one to two weeks (as disease activity allows) after each COVID vaccine dose
TNFi, IL-6R, IL-1R, IL-17, IL12/23, IL-23, and other cytokine inhibitors	No consensus
Cyclophosphamide IV	Time CYC administration so that it will occur approximately 1 week after each vaccine dose, when feasible
Hydroxychloroquine, IVIG	No modifications to either immunomodulatory therapy or vaccination timing
Rituximab or other anti-CD20 B-cell depleting agents	Discuss the optimal timing of dosing and vaccination with the rheumatology provider before proceeding
All other conventional and targeted immunomodulatory or immunosuppressive medications (e.g., JAKi, MMF) except those listed above	Hold for one to two weeks (as disease activity allows) after each COVID vaccine dose

Supplementary Table 7: Dosage of EVUSHELD for prophylaxis.

Initial dose	▪ 300 mg of tixagevimab and 300 mg of cilgavimab administered as two separate consecutive intramuscular injections.
Individuals who Initially Received 150 mg of Tixagevimab and 150 mg Cilgavimab	▪ Initial dose ≤3 months prior: 150 mg tixagevimab and 150 mg cilgavimab.<br>▪ Initial dose >3 months prior: 300 mg tixagevimab and 300 mg cilgavimab
Repeat dose	▪ 300 mg of tixagevimab and 300 mg of cilgavimab every 6 months<br>▪ Repeat dosing should be timed from the date of the most recent EVUSHELD dose

References

1. Fragoulis GE, Nikiphorou E, Dey M, Zhao SS, Courvoisier DS, Arnaud L, Atzeni F, Behrens GM, Bijlsma JW, Bohm P *et al*: 2022 EULAR recommendations for screening and prophylaxis of chronic and opportunistic infections in adults with autoimmune inflammatory rheumatic diseases. *Ann Rheum Dis* 2022.
2. Isoniazid (Rx) [https://reference.medscape.com/drug/isoniazid-342564#4]

3. Rifapentine (Rx) [https://reference.medscape.com/drug/priftin-rifapentine-342681?_ga=2.2075315.328627968.1678206610-685197622.1678206604#4]
4. Fragoulis GE, Dey M, Zhao S, Schoones J, Courvoisier D, Galloway J, Hyrich KL, Nikiphorou E: Systematic literature review informing the 2022 EULAR recommendations for screening and prophylaxis of chronic and opportunistic infections in adults with autoimmune inflammatory rheumatic diseases. *RMD Open* 2022, 8(2).
5. Park JW, Curtis JR, Moon J, Song YW, Kim S, Lee EB: Prophylactic effect of trimethoprim-sulfamethoxazole for pneumocystis pneumonia in patients with rheumatic diseases exposed to prolonged high-dose glucocorticoids. *Ann Rheum Dis* 2018, 77(5):644-649.
6. Honda N, Tagashira Y, Kawai S, Kobayashi T, Yamamoto M, Shimada K, Yokogawa N: Reduction of Pneumocystis jirovecii pneumonia and bloodstream infections by trimethoprim-sulfamethoxazole prophylaxis in patients with rheumatic diseases. *Scand J Rheumatol* 2021, 50(5):365-371.
7. Park JW, Curtis JR, Kim MJ, Lee H, Song YW, Lee EB: Pneumocystis pneumonia in patients with rheumatic diseases receiving prolonged, non-high-dose steroids-clinical implication of primary prophylaxis using trimethoprim-sulfamethoxazole. *Arthritis Res Ther* 2019, 21(1):207.
8. Morita H, Usami I, Torii M, Nakamura A, Kato T, Kutsuna T, Niwa T, Katou K, Itoh M: Isolation of nontuberculous mycobacteria from patients with pneumoconiosis. *J Infect Chemother* 2005, 11(2):89-92.
9. Vananuvat P, Suwannalai P, Sungkanuparph S, Limsuwan T, Ngamjanyaporn P, Janwityanujit S: Primary prophylaxis for Pneumocystis jirovecii pneumonia in patients with connective tissue diseases. *Semin Arthritis Rheum* 2011, 41(3):497-502.
10. Utsunomiya M, Dobashi H, Odani T, Saito K, Yokogawa N, Nagasaka K, Takenaka K, Soejima M, Sugihara T, Hagiyama H *et al*: Optimal regimens of sulfamethoxazole-trimethoprim for chemoprophylaxis of Pneumocystis pneumonia in patients with systemic rheumatic diseases: results from a non-blinded, randomized controlled trial. *Arthritis Res Ther* 2017, 19(1):7.
11. Utsunomiya M, Dobashi H, Odani T, Saito K, Yokogawa N, Nagasaka K, Takenaka K, Soejima M, Sugihara T, Hagiyama H *et al*: An open-label, randomized controlled trial of sulfamethoxazole-trimethoprim for Pneumocystis prophylaxis: results of 52-week follow-up. *Rheumatol Adv Pract* 2020, 4(2):rkaa029.
12. Harada T, Kato R, Sueda Y, Funaki Y, Takata M, Okazaki R, Hasegawa Y, Yamasaki A: The efficacy and safety of reduced-dose sulfamethoxazole-trimethoprim for chemoprophylaxis of Pneumocystis pneumonia in patients with rheumatic diseases. *Mod Rheumatol* 2021, 31(3):629-635.
13. Takenaka K, Komiya Y, Ota M, Yamazaki H, Nagasaka K: A dose-escalation regimen of trimethoprim-sulfamethoxazole is tolerable for prophylaxis against Pneumocystis jiroveci pneumonia in rheumatic diseases. *Mod Rheumatol* 2013, 23(4):752-758.
14. Suyama Y, Okada M, Rokutanda R, Min C, Sasse B, Kobayashi D, Takahashi O, Deshpande GA, Matsui K, Kawaguchi Y *et al*: Safety and efficacy of upfront graded administration of trimethoprim-sulfamethoxazole in systemic lupus erythematosus: A retrospective cohort study. *Mod Rheumatol* 2016, 26(4):557-561.
15. Bass AR, Chakravarty E, Akl EA, Bingham CO, Calabrese L, Cappelli LC, Johnson SR, Imundo LF, Winthrop KL, Arasaratnam RJ *et al*: 2022 American College of Rheumatology Guideline for Vaccinations in Patients With Rheumatic and Musculoskeletal Diseases. *Arthritis Rheumatol* 2023, 75(3):333-348.

DISCLAIMER: The above article has been published, as is, ahead-of-print, to provide early visibility but is not the final version. Major publication processes like copyediting, proofing, typesetting and further review are still to be done and may lead to changes in the final published version, if it is eventually published. All legal disclaimers that apply to the final published article also apply to this ahead-of-print version.
